# Hydrocortisone to treat early bronchopulmonary dysplasia in very preterm infants: study protocol for a randomized controlled trial

**DOI:** 10.1186/s13063-020-04698-0

**Published:** 2020-09-03

**Authors:** Yuan He, Yong Zhang, Shuqiang Gao, Xiaoling Wang, Na He, Deshuang Zhang, Wenbin Dong, Christian Wieg, Xiaoping Lei

**Affiliations:** 1 Department of Neonatology, Affiliated Hospital of Southwest Medical University, 25 Taiping Road, Luzhou, 646000 Sichuan China; 2 Department of Neonatology, Sichuan Provincial Hospital for Women and Children, Chengdu, Sichuan China; 3 Department of Neonatology, Chengdu Women’s and Children’s Central Hospital, Chengdu, Sichuan China; 4 Birth Defects Clinical Medical Research Center of Sichuan Province, Luzhou, Sichuan China; 5 Department of Perinatology, Affiliated Hospital of Southwest Medical University, Luzhou, Sichuan China; 6 Department of Neonatology, Klinikum Aschaffenburg, Am Hasenkopf 1, Aschaffenburg, 63739 Aschaffenburg, Bavaria Germany

**Keywords:** Bronchopulmonary dysplasia, Hydrocortisone, Preterm infants

## Abstract

**Background:**

Bronchopulmonary dysplasia (BPD) is still a common complication in very premature infants. At present, there is no effective treatment for BPD. Glucocorticoids are drugs commonly used to prevent or treat BPD before and after birth. In very premature infants with high risk factors for BPD, early use of dexamethasone can reduce the rate of death and/or BPD but may cause long-term adverse neurodevelopmental outcomes. Hydrocortisone (HC), as an alternative drug to dexamethasone, has been increasingly used to prevent BPD. However, no study has reported the efficacy and safety of HC to treat early BPD diagnosed at postnatal day (PND) 28.

**Methods:**

This study protocol is for a multicenter double-blind randomized controlled trial of low-dose HC in the treatment of early BPD. Early BPD infants will be randomly assigned to the HC treatment group or control group. Infants in the HC group will receive 0.5 mg/kg HC twice a day for 7 days and then 0.5 mg/kg HC once a day for 3 days. The control group will be given the same volume of placebo and no intervention on the basis of routine treatment. The primary outcome is survival without moderate or severe BPD at 36 weeks postmenstrual age. Secondary outcomes are the short- and long-term effects on growth, metabolism, neurodevelopment, and other possible complications.

**Discussion:**

This trial will determine the efficacy and safety of low-dose HC administration compared to placebo for the reduction of moderate or severe BPD at 36 weeks postmenstrual age in very preterm infants with early BPD.

**Trial registration:**

China Clinical Trial Registration Center ChiCTR1900021854. Registered on 13 March 2019.

## Background

Bronchopulmonary dysplasia (BPD) is a serious complication in very preterm infants, and the incidence has been no significant decrease in the past two decades [1]. When BPD is present in preterm infants, mortality and short- and long-term comorbidities are increased, and the need for health and education services is required more frequently than non-BPD infants later in life [2]. Since BPD was first described by Northway in 1967, the understanding of the pathophysiology evolved, and the definitions of BPD changed over time [3]. In 2001, the National Institute of Child Health and Human Development (NICHD) workshop defined BPD as the need for supplemental oxygen on the 28th day of life and categorized it as “mild, moderate, and severe” at 36th week postmenstrual age (PMA) (or 56 days of age for infants > 32 weeks GA), based on oxygen use and/or respiratory support [4]. Although this definition is widely used, its prognostic value is limited due to its inconsistent correlation with long-term respiratory outcomes and the inability to classify infants dying from severe respiratory failure prior to 36 weeks of PMA [5]. Thus, the Vermont-Oxford Network modified the BPD definition to need for oxygen supplementation at 36th week PMA [6, 7], perceiving a high accuracy in predicting long-term respiratory outcomes but being unable to guide the early treatment of BPD.

Inflammation plays a key role in the pathogenesis of BPD by providing the rationale for treating BPD with glucocorticoids, which have potent anti-inflammatory effects [8, 9]. A large number of studies have shown that glucocorticoids, especially dexamethasone, improve short-term pulmonary function in evolving BPD, but there is no evidence for beneficial long-term pulmonary outcomes. Since the reports by Shinwell et al. [10], concerns regarding adverse neurodevelopmental outcomes of premature infants exposed early to dexamethasone have increased [11, 12]. Supported by a systematic review, the actual conclusion of this debate is that the short-term benefits of postnatal dexamethasone do not outweigh the long-term adverse effects. Thus, dexamethasone is recommended only for infants who cannot be weaned from mechanical ventilation more than 7 days after birth [11–13].

As an alternative glucocorticoid, hydrocortisone (HC) has been considered to replace dexamethasone [14, 15]. In newborn rats, HC had no detrimental effect on neurodevelopment [16], and infants exposed to HC in neonatal life had a higher median motor optimality score at the age of 3 months compared to those with dexamethasone exposure [17]. Furthermore, some studies have indicated that exposure to low-dose HC (1–2 mg/kg/d) leads to increased survival without BPD [9, 18–21] and does not increase adverse long-term neurodevelopmental sequelae [22–25]. However, these studies only used HC as a prophylactic drug (started at ≤ 7 days or between 7 and 14 days after birth) [9, 18–26].

In current clinical practice, according to the NICHD criteria, 68–77% of very preterm infants are diagnosed with BPD at 28 days of life (early BPD), while approximately 60% of early BPD infants still need oxygen or respiratory support at a PMA of 36 weeks (moderate or severe BPD) [27–29]. Thus, it is essential to find an effective and safe drug to prevent early BPD from developing to moderate or severe BPD. However, based on the current recommendations, except those needing mechanical ventilation, very preterm infants will not be treated with glucocorticoids at this stage. Based on the use of early low-dose HC in infants with a high risk of BPD in previous studies [9, 18–25], an RCT study is reasonable to evaluate the efficacy and safety of low-dose HC in treating early BPD.

## Study design and methods

### Aim of the study

To investigate the efficacy and safety of a low dose of HC in the treatment of early BPD, diagnosed at postnatal day (PND) 28 in preterm infants.

### Study design

This multicenter double-blind randomized controlled trial will be performed at three centers in China. The enrollment period will be 1 year, and there will be a series of follow-up for every child until 2 years corrected age, which is the revised age from the 40th gestation week for a premature baby. The human research ethics committees at each participating center approved the trial, and an independent academic board will monitor the trial.

### Participants

Inclusion criteria:

1. Gestational age 26^+0^ to 30^+6^ weeks and birth weight < 1500 g; and
2. Diagnosed early BPD at day 28, based on NICHD criteria [4] and never received any glucocorticoids after birth; and
3. One of the following conditions:
  Oxygen reduction test showing SpO_2_ < 90% with FiO_2_ > 0.3 or
  Noninvasive ventilation support or nasal cannula with FiO_2_ > 0.21.

Exclusion criteria included any of the following conditions:

1. Invasive ventilation support needed on the 28th day of life.
2. Congenital malformations affecting pulmonary function (e.g., congenital surfactant protein deficiencies, congenital diaphragmatic hernia) or increasing the risk of death or adverse neurodevelopmental outcome (e.g., congenital cerebral malformations, congenital hydrocephalus).
3. Chromosomal defects (e.g., trisomy 13, 18, 21).
4. Severe infection less than 7 days before randomization (late-onset sepsis/meningitis confirmed by positive blood culture; bacterial pneumonia, confirmed by positive tracheal culture; neonatal necrotizing enterocolitis).

### Sample size calculation

According to the data from the participating centers in the past 2 years and the data presented from the range of previous studies [27–29], the incidence of early BPD in infants with gestational age 26^+0^ to 30^+6^ weeks was 68–77%, and 40% developed moderate or severe BPD. The effect of HC therapy was assumed to reduce moderate or severe BPD (the primary endpoint) to 25% or less (number needed to treat, NNT = 5). Adopting a maximum significance level of *α* = 0.05 and a minimum test power of 80%, the number of subjects per group will be *N* = 81, which will be increased by 20% to compensate for potential dropouts during the follow-up, leading to 98 subjects per group (total 98 × 2 = 196 subjects).

### Trial medication and dosing

Trial medication will be prepared and distributed according to Good Clinical Practice (GCP) and Good Manufacturing Practice (GMP) guidelines. Using a double-blinded, placebo-controlled design, infants will be allocated to either HC or placebo treatment.

The long-term adverse neurodevelopmental outcomes observed with dexamethasone use are associated with the accumulative dosage [30]. The optimal (cumulative) dose of HC is currently unknown. In one previous study, Baud et al. [21] found that an early (< 7 days) low dose of HC increased the rate of survival without BPD at 36 weeks PMA without adverse neurodevelopmental outcomes at 2 years of age [22, 24]. Based on these findings, we adopted this dosage in the present study. Infants randomly assigned to the HC group will receive 0.5 mg/kg HC twice per day for 7 days, followed by one dose of 0.5 mg/kg per day for 3 days. This leads to a total duration of therapy of 10 days and a cumulative dose of 8.5 mg/kg HC. The control group will receive an equal volume of saline and will not receive any other intervention on the basis of routine treatment.

### Use of cointervention

All randomized patients will be subjected to cointerventions, such as respiratory support and comedication, according to the guidelines and protocols of the participating centers. The participants will not be given other treatments outside of the trial interventions and cointerventions.

### Informed consent

Professional researchers will carefully introduce the detailed procedures, probable benefits, and potential risks of the study to the guardian of the infants who meet the inclusion criteria. The infants’ guardians will indicate their willingness to participate voluntarily by signing an informed consent form.

### Randomization

Eligible patients will be randomized at day 28 after birth in each center after at least one guardian provides informed consent. A list of all eligible patients not included in the study will be provided from all centers throughout the time of enrollment. This trial will be protected from selection bias by using concealed, stratified, and blocked randomization. Randomization will be stratified according to gestational age stratum (26^+0^ to 28^+6^ weeks, and 29^+0^ to 30^+6^ weeks) in each study center. Multiple birth infants will be internally randomized. The randomization procedure will be generated by using Excel software by the GCP faculty at the participating hospitals, and it will be sealed in a sequentially numbered envelope to maintain the allocation concealment. According to the allocation sequence, drug packages will be hidden and labeled sequentially. Participants will receive packaged drug in the order of recruitment. The first dose of study medication will be administered within 1 h after randomization.

The complete random sequence will be kept by the primary researcher at the affiliated hospital of Southwest Medical University. Centers will individually have emergency envelopes that contain the treatment information for patients. Emergency envelopes can be unsealed only under special conditions.

### Outcomes

#### Primary outcome

The primary outcome is survival without moderate or severe BPD at PMA 36 weeks, which is defined as the following criteria: no respiratory support, spontaneous breathing FiO_2_ 0.21 for SpO_2_ 92%.

#### Secondary outcomes

1. Amplitude-integrated electroencephalography (aEEG). A series of aEEGs will be monitored once a week for all preterm infants after 30 weeks PMA. Sleep-wake cycles of aEEG can be classified as absent, mature, or immature according to Hellstrom-Westas [31]. The PMA of mature sleep-wake cycles occurred will be used as one of the neurodevelopmental outcomes and be compared between two groups.
2. Intraventricular hemorrhage (IVH) > grade II and/or periventricular leucomalacia (PVL). A series of cranial ultrasound scans will be performed regularly for all preterm infants, and these results will be extracted from the medical records after enrollment. IVH > grade II and/or PVL will be used as an adverse outcome to be compared between the groups [32].
3. Brain volume at 40 weeks PMA. Total brain volume will be detected by using the functional MRI of the brain Software Library for automatic segmentation of the T1/T2 weighted images. The mean/median total brain volumes will be compared between groups at 40 weeks.
4. Nosocomial infection, including cultured proven sepsis, meningitis, and pneumonia. Culture-proven infection is defined as a positive culture obtained from sputum, urine, blood, or cerebrospinal fluid of infants with clinical signs of infection, and pneumonia will be confirmed by abnormal lung examination (such X-ray, CT).
5. Neonatal necrotizing enterocolitis (NEC). Bell stage II or more and/or focal intestinal perforation diagnosed by X-ray after enrollment to hospital discharge [33].
6. Gastrointestinal bleeding diagnosed by the contents of the digestive tract.
7. Retinopathy of prematurity (ROP). A series of fundus examinations will be arranged after enrollment, and ROP that needs treatment in any stage will be used as an adverse outcome [34].
8. Anthropometric indices. Body length, head circumference, and body weight at 36 weeks of PMA.
9. Hyperglycemia. Hyperglycemia that needs to be treated with insulin (serum glucose levels ≥ 200 mg/dl) after enrollment.
10. Hospitalization days.

#### Long-term outcomes after hospital discharge

1. Neurodevelopmental impairment at 104 weeks corrected age: The Bayley Scales of Infant and Toddler Development, Second Edition (BSID II) will be used in this trial.
  - Cognitive delay: defined as a cognitive score less than 80.
  - Language delay: defined as a language score less than 80.
  - Cerebral palsy and severity of cerebral palsy using the gross motor function classification system.
2. Hearing loss requiring hearing aids at 104 weeks corrected age.
3. Blindness at 104 weeks corrected age: vision loss with only form or shadow vision or no useful vision.
4. Number of readmissions in the first year since the first discharge home.
5. Weight, length, and head circumference at 40 weeks postmenstrual age and 12, 26, 52, and 104 weeks corrected age.
6. Premature birth-related mortality from enrollment to 104 weeks corrected age.
7. Behavioral problems (child behavior checklist) at 104 weeks corrected age. The scores on the checklist will be compared between groups.

### Clinical data collection

At the time of randomization, baseline data will be collected from all included patients, including maternal and patient characteristics. Demographic data will include sex, birth weight, gestational age, 5-min Apgar score, surfactant therapy, prenatal corticosteroid use, premature rupture of the membrane before the onset of labor (> 18 h), singleton/multiple birth, and mode of delivery. Population characteristics will be used to compare outcomes between the two groups.

The following clinical data will be collected: continuous positive airway pressure (CPAP)-assisted time, invasive ventilator-assisted time, days of hospitalization, days on supplemental oxygen, IVH, spontaneous intestinal perforation, NEC, and ROP.

During HC treatment, respiratory support, blood sugar, blood pressure, and anthropometric data will be collected. Outcome variables and cointerventions will be recorded at 36 weeks PMA.

After discharge, one trained investigator in each center will follow up all patients at 40 weeks PMA and at 12, 26, 52, and 104 weeks corrected age. We will provide free neurodevelopmental impairment examinations to promote follow-up rates. For potential dropouts during the follow-up, we will expand the sample size by 20% and the missing data will be ignored in the analysis. The outcomes at each time point mentioned above will be collected by the investigators (Table 1).

**Table 1:** SPIRIT figure: showing time points for enrollment, interventions, and assessment

	Study period						
	Enrollment	Allocation	Post-allocation				Close-out
Time point	At 4 weeks after birth	At 4 weeks after birth	At 36 weeks postmenstrual age	At 40 weeks postmenstrual age	At 12, 26 weeks corrected age	At 52 weeks corrected age	At 104 weeks corrected age
Enrollment:							
Eligibility screen	X						
Informed consent	X						
Demographic data	X						
Allocation		X					
Intervention:							
Hydrocortisone		X					
Placebo		X					
Assessments:							
Baseline variables assessments	X	X					
Primary outcome			X				
Secondary outcomes:							
Brain volume				X			
Others			X				
Long-term outcomes:							
Neurodevelopmental impairment, hearing loss, and blindness							X
Number of readmissions						X	
Weight, length, and head circumference				X	X	X	X
Behavioral problems, premature birth-related mortality							X

### Data management

All documents and source documents will be stored in the data center. These documents include informed consent, all reports, administrative forms, copies of medical records, and other identifiable patient material. In the data center, data will be stored in file cabinets locked to areas with limited access, thus keeping participants anonymous. All electronic participant data will be deposited in our department’s relevant platform. The principal investigator will have access to the final data sets and be responsible for the data. After application and permission, related researchers can have access to the anonymous data for future studies. Additionally, we will deliver a completely deidentified data set to an appropriate data archive for sharing purposes.

### Statistical analysis

The analyses will be based on the intention-to-treat (ITT) principle in accordance with the Consolidated Standards of Reporting Trials (CONSORT). Statistical analyses will be performed with SAS version 9.2 (SAS Institute, Cary, North Carolina). Data will be presented as the mean ± standard deviation or median and interquartile range depending on their distribution. Categorical data, including the primary outcome, will be analyzed using the chi-square test between two groups. Continuous data will be analyzed using Student’s *t* test or the Mann-Whitney test as appropriate. The effect of HC on the dichotomous outcomes will be assessed by multivariable logistic regression analysis including possible confounders, including birth weight and gestational age. Statistical results will be reported as point estimates and 95% confidence intervals.

Mid-term data analysis will be conducted after inclusion of the first 98 patients. The principal investigator will decide whether any measure should be taken in light of the results. Case report forms (CRFs) will be entered by two independent researchers to ensure accuracy.

### Ethical and academic management

The research protocol was reviewed and approved by the Ethics Committee of Southwest Medical University, and we will not begin recruiting at other centers in the trial until local ethical approval has been obtained. The ethics committee will be responsible for supervising all procedures of the study. Prior to making any changes to the study protocol, a written application will be submitted to the ethics committee for approval, and new written informed consent will be obtained from the guardians.

An independent academic board, which is composed of competent department, data monitoring, and safety management advisors, has been implemented to oversee unwanted effects and risks, as well as the collection and management of the data. The academic board will review all research data every 3 months. Until the included patients are discharged, each potential adverse effect will be monitored and reported to the academic board within 48 h. All members of the academic board are independent of the sponsor and trial investigators and free of competing interests.

Participants, as well as the researchers, evaluators, doctors, and nurses who are responsible for screening, recruitment, dispensing medicine, and outcome assessment, will remain blinded to treatment allocations. The researchers conducting the statistical analysis will know only the group codes but not the corresponding interventions. When severe adverse effects occur, the academic board has the right to unseal emergency envelopes. The reason for unblinding and time will be recorded. We prepared an insurance policy for every participant. This will provide ancillary and posttrial care in the case of any harm resulting from participation in the clinical trial.

After consulting the academic board, the treatment will be suspended for the following conditions: (1) serious adverse reactions (death or severe infections) that occur during the testing or (2) a guardian request withdraw their infant from the study at any time and without any consequences. The primary outcome and other data collected for participants until the date of withdrawal will be used and analyzed as part of the ITT population.

Patients and the public were not involved in the design of the present study. The results of the trial will be published in a peer-reviewed journal and will be presented at national and international conferences. The final author’s confirmation will be based on the contribution to the study and there are no plans to utilize professional writers.

### Quality control

To ensure a full understanding and mastery of the standard operating procedures, all researchers will be trained before the trial begins. Independent investigators at the Affiliated Hospital of Southwest Medical University will regularly monitor the quality of the data collected on the CRFs. If any error exists, researchers will be notified immediately.

## Discussion

In babies < 26^+0^ weeks gestational age, several problems, such as PDA, severe apnea, and high thorax compliance, may lead to noninvasive respiratory dependency at day 28 without any link to BPD. Furthermore, a higher rate of sepsis was observed in infants born at 24–25 weeks gestational age who were treated with hydrocortisone in a previous study [21]. Therefore, we will restrict the eligible patients to those between 26^+0^ and 30^+6^ weeks gestational age.

Because inflammation plays a key role in BPD, postnatal glucocorticoids are thought to be the most effective therapeutic strategy for preterm infants with a high risk for developing BPD [35], but they are accompanied by severe long-term adverse neurodevelopmental outcomes [11, 12]. These adverse outcomes seem to be associated with the timing of steroid exposure and the cumulative dosage of glucocorticoids; the earlier the treatment initiation and/or the larger the dosage, the higher the risk of adverse outcomes [11, 15].

Following the actual recommendations, many infants presenting with early BPD do not receive any anti-inflammatory therapy, and a high percentage of premature infants are still oxygen-dependent or on respiratory support at 36 weeks PMA (moderate or severe BPD) [27–29], which is associated with poor long-term pulmonary outcomes, including home oxygen therapy and a high rate of bronchopulmonary infections and rehospitalization [2]. A recent study showed that moderate or severe BPD at 36 weeks PMA was associated with increased mortality in the first year of life and moderate to severe neurodevelopmental impairment (55%) [6]. Thus, we hypothesize that anti-inflammatory treatment of infants meeting the NICHD criteria for BPD at 28 days of life may decrease the incidence of moderate or severe BPD at 36 weeks PMA and reduce long-term adverse outcomes.

In previous studies, early low-dose HC therapy in ventilator-dependent infants improved the rate of survival without BPD at 36 weeks PMA [21] without adverse neurodevelopmental outcomes at 2 years of age [22, 24]. Thus, it seems reasonable to use low-dose HC to treat infants with early BPD to improve pulmonary outcome. Accordingly, the efficacy and safety of this treatment must be proven by a randomized controlled trial.

## Trial status

Data collection will begin from August 1, 2019, and will end in July 31, 2022. The protocol version number and date are as follows: Second Edition, 13 March, 2019.

## Data Availability

The authors declare that all relevant data will be included in the article or supplementary files. Additional data are available from the corresponding author on reasonable request.

## Abbreviations

BPD: Bronchopulmonary dysplasia
PND: Postnatal day
NICHD: National Institute of Child Health and Human Development
PMA: Postmenstrual age
GA: Gestational age
HC: Hydrocortisone
RCT: Randomized controlled trial
GCP: Good Clinical Practice
GMP: Good Manufacturing Practice
IVH: Intraventricular hemorrhage
PVL: Periventricular leucomalacia
NEC: Neonatal necrotizing enterocolitis
ROP: Retinopathy of prematurity
CPAP: Continuous positive airway pressure
ITT: Intention to treat
CONSORT: Consolidated Standards of Reporting Trials
CRFs: Case report forms
